# Customized treatment for an oncologic lesion near a joint: case report of a custom-made 3D-printed prosthesis for a grade II chondrosarcoma of the proximal ulna

**DOI:** 10.1016/j.jseint.2020.07.008

**Published:** 2020-08-12

**Authors:** Annemarie S.E. Brandsma, Egbert Jan D. Veen, Andor W.J.M. Glaudemans, Paul C. Jutte, Joris J.W. Ploegmakers

**Affiliations:** a Department of Orthopedic Surgery, University Medical Center Groningen, Groningen, the Netherlands; b Department of Nuclear Medicine, University Medical Center Groningen, Groningen, the Netherlands

**Keywords:** Chondrosarcoma, ulna, 3D-printed prosthesis, orthopedic surgery, oncology

The first goal in orthopedic oncology is resection with a free margin while preserving as much function as possible. Techniques popularized in recent decades with the potential to advance reconstruction and thus function following resection include allograft transplantation and prosthetic replacement. Every operative technique has its limitations.

New solutions can be pioneered thanks to the development of prosthetic printing technology (both 3D-printed patient-specific instruments^8^ and custom-made 3D-printed prostheses^2^^,^^5^^,^^7^^,^^13^^,^^14^). These printed prostheses have the potential for a perfect fit aimed at restoring function.

We present a case in which a personalized 3D-printed custom-made proximal ulna was used as hemiarthroplasty to reconstruct the ulna after resection of a chondrosarcoma, thereby sparing function and avoiding a complete elbow prosthesis. Shown are the successful functional and ingrowth results after 2.5 years' follow-up with preservation of the distal humerus and humeroradial joint.

## Case report

In 2008, a 42-year-old fit and healthy man sustained a pathologic fracture (suspected of cartilaginous lesion) of the left olecranon. The patient was treated with curettage, autologous bone grafting, and fixation with plate and screws. Pathologic examination confirmed the diagnosis of chondrosarcoma grade I. Rehabilitation went uneventfully, as did removal of hardware in 2009.

**Figure 1:**
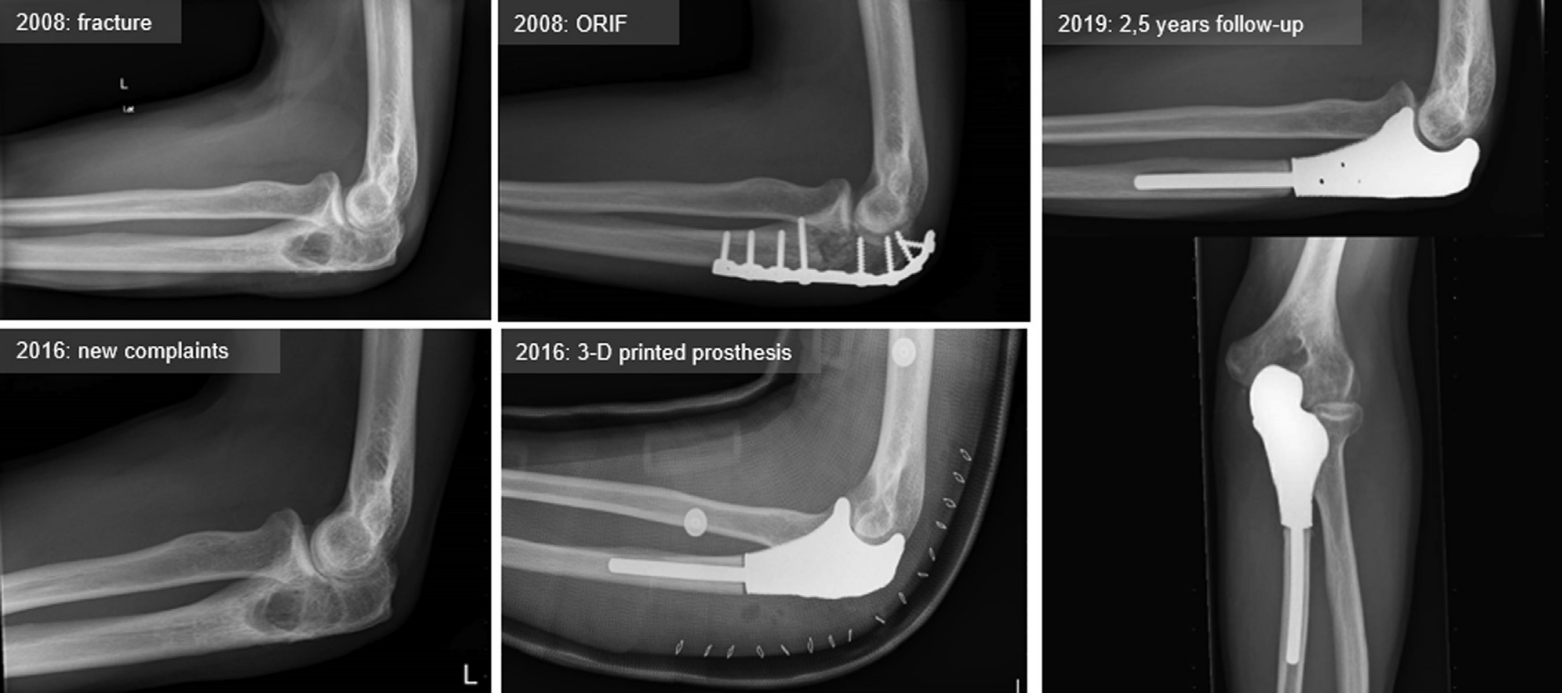
Timeline. *ORIF*, open reduction and internal fixation.

**Figure 2:**
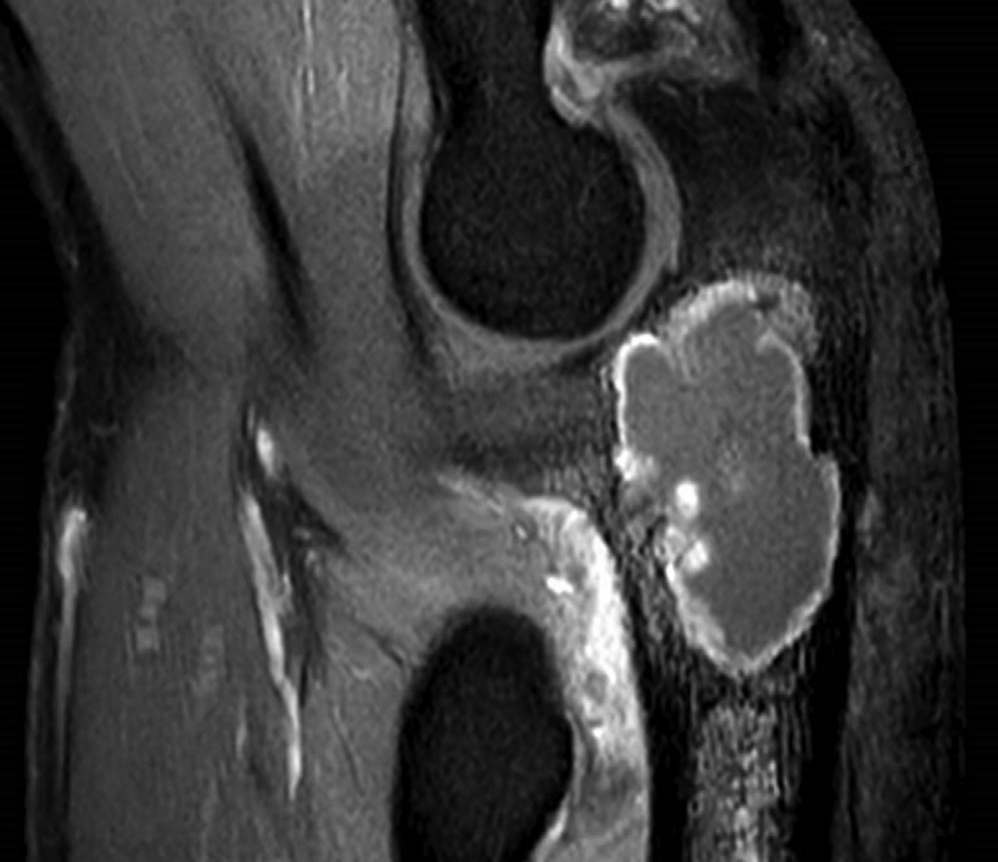
Magnetic resonance imaging scan with gadolinium contrast.

Seven years later, in 2016, at the age of 49, the patient was referred to our orthopedic oncology center with nocturnal pain in his elbow joint. Physical examination showed a limited extension of 40° and a palpable mass at the left olecranon. Figure 1 depicts a radiographic timeline of the ulna. Radiographic examination and magnetic resonance imaging with gadolinium contrast revealed an expansive lytic lesion of the proximal ulna with clear scalloping and some perilesional edema, which raised the suspicion of recurrence of a more aggressive type of chondrosarcoma (Fig. 2). Histologic evaluation by computed tomography (CT)-guided biopsy showed signs of a grade II chondrosarcoma in the proximal ulna with preservation of the cortex. According to the World Health Organization, grade I chondrosarcoma is classified as an atypical cartilaginous tumor. In the absence of mitosis, the atypical cartilaginous tumor is unlikely to metastasize and is thereby considered to be a locally aggressive neoplasm rather than a malignant sarcoma. Grade II chondrosarcoma, however, acts more aggressively and has an intermediate risk of metastasizing (10%-20%)^1^ as well as histologic features of hypercellularity with pleomorphisms, extension of myxoid matrix components, and invasive tumorous ingrowth in trabecular bone.

Dissemination studies turned out to be negative. In conclusion, radiographs, magnetic resonance imaging, and histologic evaluation pointed to a diagnosis of grade II chondrosarcoma. In case of a recurrent grade I chondrosarcoma (atypical cartilaginous tumor), a recurettage would be adequate treatment. However, with a recurrent and apparent histologic grade II chondrosarcoma, rigorous treatment using wide excision is necessary to achieve clean margins.^16^^,^^20^ The patient was planned for marginal resection of the tumor and reconstruction with a novel hemiarthroplasty technique using a custom-made proximal ulna, the 3D-printed prosthesis type MUTARS (Modular Universal Tumor And Revision System; implantcast, Buxtehude, Germany).

### Preoperative planning

**Figure 3:**
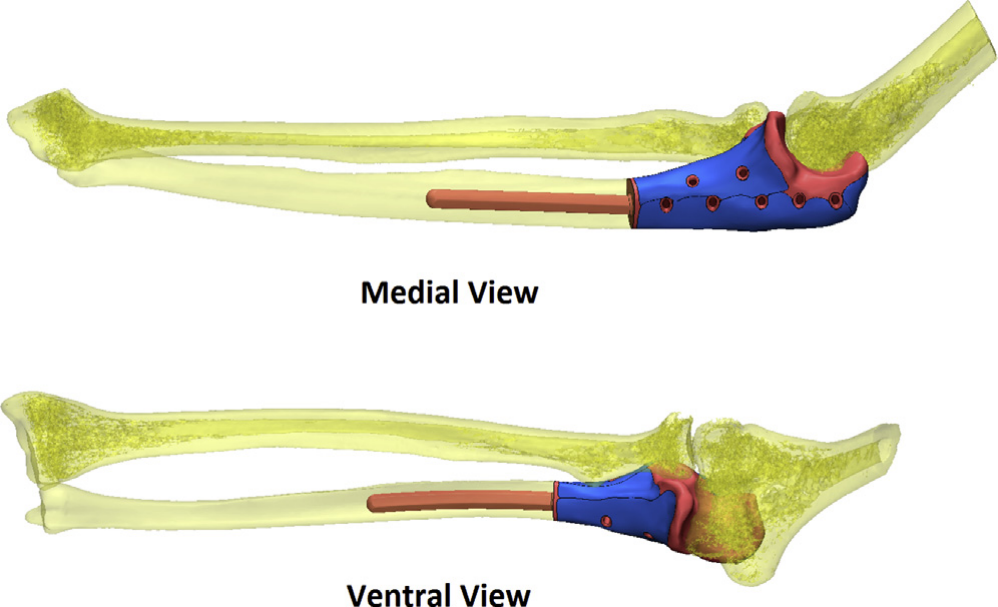
3D computer-aided design planning of trial prosthesis.

**Figure 4:**
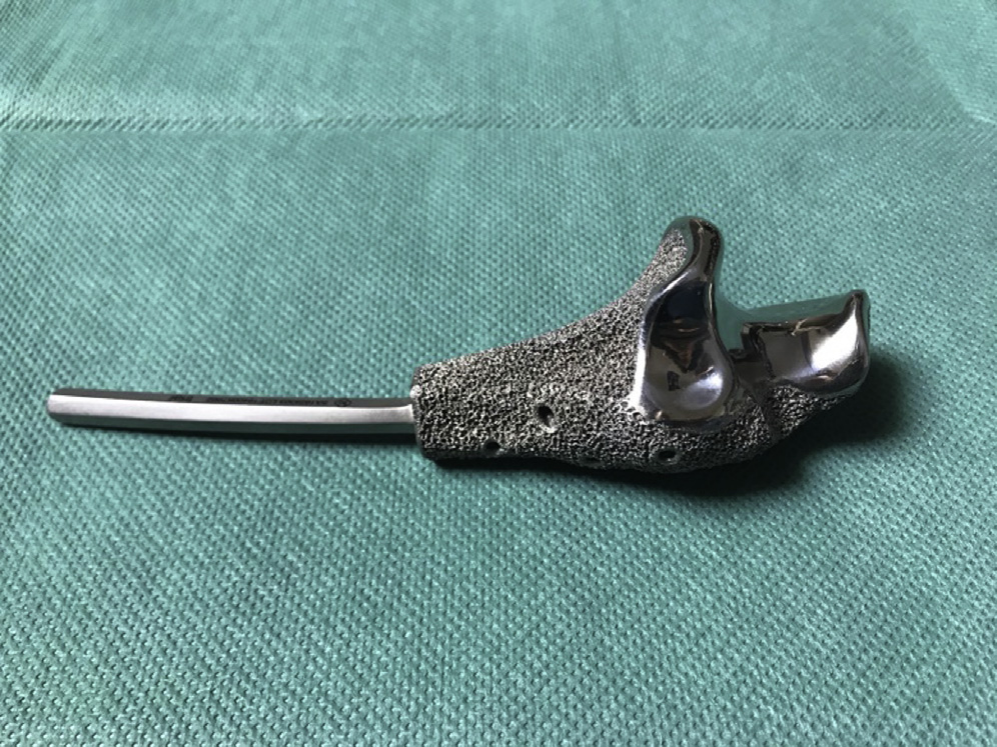
3D-printed model of trial prosthesis.

Based on CT scan of the bony structures of the ipsilateral elbow, the resection was planned and a computer-aided design model for the implant was made, including especially designed additional fixation holes for the triceps tendon and collateral ligaments (Fig. 3). Next, the implant was printed by an electron-beam melting technique with titanium powder. Once printed, the implant was milled, polished, and coated with additional titanium nitride (Fig. 4).

### Surgical technique

#### Preparation and dissection of the elbow

The patient was placed in a lateral decubitus position with the left arm resting over a roll. The relevant anatomy of the elbow and the scar of the previous incision were marked over the olecranon. Prophylactic antibiotics were administered intravenously. After standard disinfection and preparation, a longitudinal incision over the proximal ulna and distal humerus was made.

The ulnar nerve was released and kept intact. The distal triceps tendon was detached from the ulna, including the periosteal sleeve and anconeus fascia, to preserve the triceps tendon for reinsertion into the prosthesis. The proximal radial head could be preserved with its annular ligament. Lastly, the humeral articulation was released.

### Resection of the tumor

The next step was en bloc resection of the grade II chondrosarcoma with the cut of the ulna based on the magnetic resonance imaging scan in order to have enough distance from the tumor and achieve adequate margins. A cut was made with a saw 8 cm distally from the olecranon tip, and the specimen was sent for histologic examination.

### Preparation of the ulna

The ulnar shaft was reamed to create a 5-mm-diameter hole of sufficient depth. A 3D-printed trial implant was seated and fitted correctly, with a focus on restoring length, rotation, and articulation with the radius and ulna.

### Implantation

Next, the definitive implant was impacted press-fit with the right rotation. The accessory holes in the prosthesis were used to attach the triceps with its periosteal layer and the medial ulnar collateral ligaments using nonresorbing sutures. After reattachment, the elbow felt stable. The ulnar nerve was placed in a submuscular tissue envelope but not purposefully transposed. The subcutaneous and skin layers were closed over the prosthesis without tension. A bandage and shoulder-elbow immobilizer were placed on the arm. The immediate postoperative radiograph showed an adequate position of the prosthesis. The motor and sensory function of the medial, radial, and specifically the ulnar nerve remained intact directly postoperatively, with a slight sensory loss in the fifth digit.

### Postoperative management

The sling was maintained for 6 weeks of complete immobilization, after which physiotherapy was started for passive motion extension/flexion and pronation/supination.

### Follow-up

**Figure 5:**
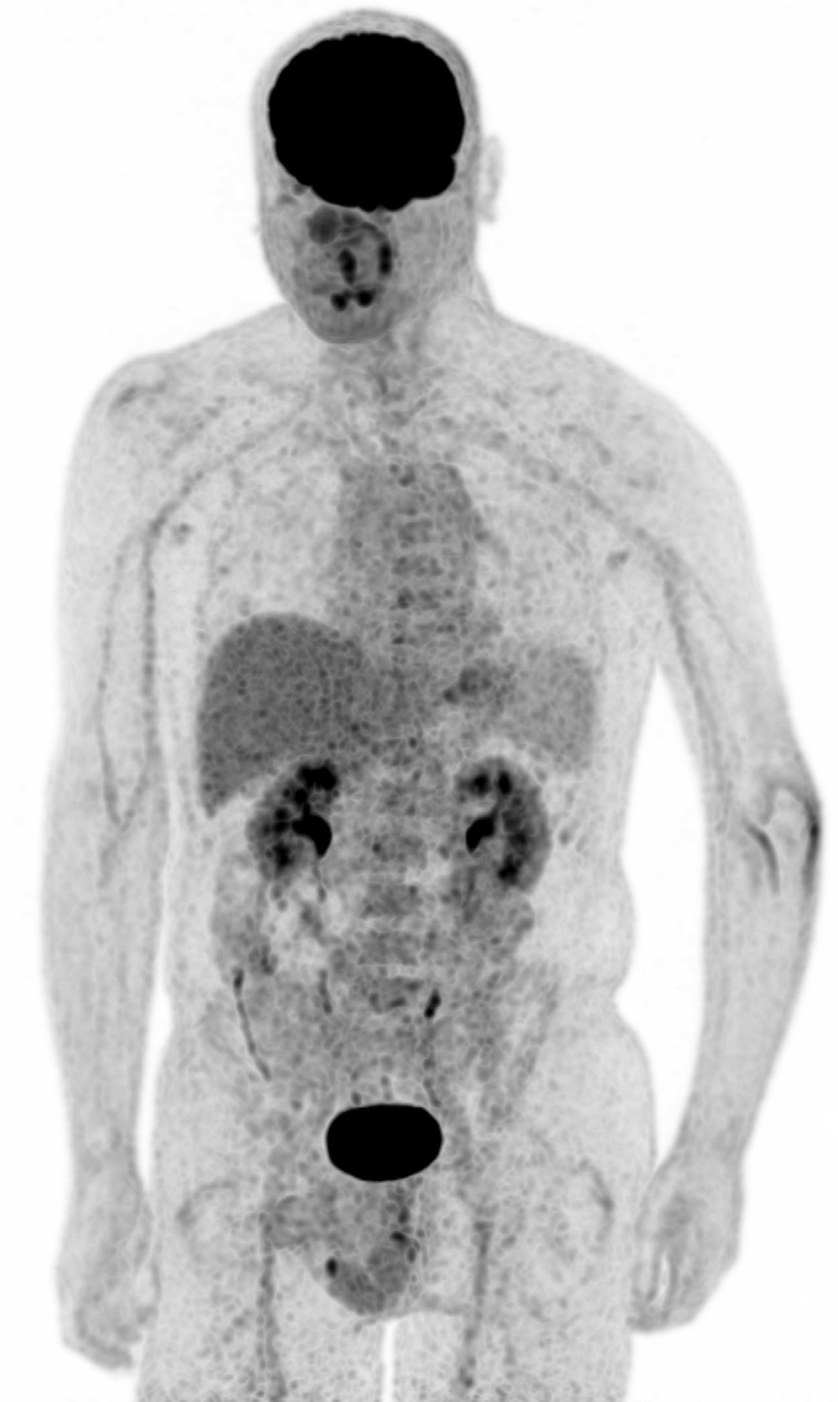
Technetium FDG PET/CT scan at 2.5 years postoperation.

Pathology report confirmed the diagnosis of grade II chondrosarcoma with wide margins of 25 mm to resection plane and 5 mm distance to the joint. In the following months, the patient recovered painlessly, with 140/10° flexion/extension and 80/50° pronation supination at the 18-month follow-up, and 140/30° flexion/extension and 90/70° pronation/supination at 2 years. Elbow function remained the same at 2.5 years' follow-up. Conventional radiographs (Fig. 1) showed an adequate position of the prosthesis with no signs of loosening or wear of the remaining joint although a developing lucent zone is seen at the distal anterior zone of the ulnar stem. FDG PET/CT scan confirms the absence of loosening (Fig. 5). Active extension of the elbow is still limited at 10° (probably due to a limited triceps ingrowth on the prosthesis), with strong flexion including a stable radial head in external rotation and only slight medial and lateral ulnar collateral instability in varus and valgus, though without limitations in daily life. The patient is able to perform his job as a clerk and now does weightlifting and works overhead with his nondominant and nonaffected arm. Assessment with a Disabilities of the Arm, Shoulder, and Hand (DASH) questionnaire gave a score of 25.8 points (standardized scale from 0 [no disability] to 100).^8^ The slight sensitivity loss in his fifth digit persisted. 18F-fluorodeoxyglucose (FDG) positron emission tomography revealed no signs of recurrent tumor or metastases. The accompanying low-dose CT scan showed an adequate position of the prosthesis with good ingrowth into the ulna, without signs of osteoarthritis of the distal humerus.

## Discussion

Reconstruction with a personalized 3D-printed custom-made proximal ulnar hemiarthroplasty is reported after resection of a chondrosarcoma grade II of the proximal ulna in a 49-year-old man. Follow-up at 2.5 years shows fair elbow function with prosthetic ingrowth. To our knowledge, this is the first case report describing a solitary 3D-printed proximal ulnar prosthesis.

When performing oncologic surgery, the challenge lies in complete resection of the tumor while preserving function by sparing as much bone, ligament, capsule, tendon, and muscle as is oncologically safe. Various reconstructive methods have been developed over the years: allograft, autograft, and tumor prosthesis surgery, all with advantages and limitations. Allograft may provide excellent support, yet there is the possibility of resorption and nonunion. Another daunting limitation of allograft is the lack of long-term incorporation, often restricted to the interface.^12^^,^^9^ Autograft has excellent healing capacity but also has donor site morbidity and a limited supply in case of a large defect. Prosthetic tumor surgery may provide immediate support and fast return to function, yet it is associated with wound infection, loosening, and persistent pain. Furthermore, these types of prostheses have a limited life span.^3^^,^^6^^,^^17^

Recent literature evidences the successful use of 3D-printed models, making patient-specific instruments^11^ when resecting bones for prosthetic reconstruction or osteotomies and 3D-printed implants to restore the anatomy of massive bony defects.^2^^,^^10^ 3D-printed implants could be particularly successful for metaphyseal bone, for example, in the proximal tibia, and still do not involve the articular surface of the joint.^10^^,^^21^

In our case there were several reasons to choose a hemiarthroplasty over other options: because of the large size and high grade of the tumor, en bloc excision had to be performed near the elbow joint. As a consequence, replacement of an articular surface would be insufficient to reconstruct with a vascularized fibular graft because of insufficient fit, and fixation could be problematic for vascularization. Second, because the tumor had invaded only into the proximal ulna, a total elbow prosthesis would result in unnecessary sacrifice of the humeroulnar joint. Total elbow arthroplasties have shown varying results in terms of survival,^4^^,^^18^ especially in patients younger than age 50 years.^15^

Both custom-made 3D templates and implants have shown promising result in lower- and upper-extremity osteoarthritis,^2^^,^^5^^,^^13^^,^^14^^,^^19^ and likewise in treating bone tumors. Several of these novel techniques have proven beneficial in oncologic bone surgery.

Potential flaws of a custom-made 3D-printed prosthesis are unrestored rotation, chances of humeral erosion, and overstuffing. The scarce literature shows only short-term results, making long-term follow-up essential.

## Conclusion

This case demonstrates use of a custom-made 3D-printed prosthesis of the proximal ulna for the treatment of a grade II chondrosarcoma. Satisfactory function is seen at the 2.5-year follow-up. Use of 3D-printed prostheses can be a valuable adjunct for oncologic orthopedic surgeons when treating bony tumors near a joint.

## Disclaimer

The authors, their immediate families, and any research foundations with which they are affiliated have not received any financial payments or other benefits from any commercial entity related to the subject of this article.
